# Human Adenovirus Serotype 5 Is Sensitive to IgM-Independent Neutralization In Vitro and In Vivo

**DOI:** 10.3390/v11070616

**Published:** 2019-07-05

**Authors:** Andor Doszpoly, Fernando de la Cuesta, Estrella Lopez-Gordo, Cécile Bénézech, Stuart A. Nicklin, Andrew H. Baker

**Affiliations:** 1 Centre for Cardiovascular Science, Queen’s Medical Research Institute, University of Edinburgh, Edinburgh EH16 4TJ, UK; 2 Institute of Cardiovascular and Medical Sciences, BHF Glasgow Cardiovascular Research Centre, University of Glasgow, Glasgow G12 8TA, UK

**Keywords:** human adenovirus 5, virus neutralization, immune response, mouse IgM, complement components

## Abstract

Human adenovirus 5 (HAdV-5) is used as a vector in gene therapy clinical trials, hence its interactions with the host immune system have been widely studied. Previous studies have demonstrated that HAdV-5 binds specifically to murine coagulation factor X (mFX), inhibiting IgM and complement-mediated neutralization. Here, we examined the physical binding of immune components to HAdV-5 by nanoparticle tracking analysis, neutralization assays, mass spectrometry analysis and in vivo experiments. We observed that purified mouse Immunoglobulin M (IgM) antibodies bound to HAdV-5 only in the presence of complement components. Active serum components were demonstrated to bind to HAdV-5 in the presence or absence of mFX, indicating that immune molecules and mFX might bind to different sites. Since binding of mFX to HAdV-5 blocks the neutralization cascade, these findings suggested that not all complement-binding sites may be involved in virion neutralization. Furthermore, the data obtained from serum neutralization experiments suggested that immune molecules other than IgM and IgG may trigger activation of the complement cascade in vitro. In vivo experiments were conducted in immunocompetent C57BL/6 or immuno-deficient *Rag2^-/-^* mice. HAdV-5T* (a mutant HAdV-5 unable to bind to human or mFX) was neutralized to some extent in both mouse models, suggesting that murine immunoglobulins were not required for neutralization of HAdV-5 in vivo. Liquid Chromatography-Mass Spectrometry (LC-MS/MS) analysis of HAdV-5 and HAdV-5T* after exposure to murine sera showed stable binding of C3 and C4b in the absence of mFX. In summary, these results suggest that HAdV-5 neutralization can be mediated by both the classical and alternative pathways and that, in the absence of immunoglobulins, the complement cascade can be activated by direct binding of C3 to the virion.

## 1. Introduction

Human adenoviruses have been used as gene therapy vectors for the past four decades. Adenoviral vectors have large DNA packaging capacity (7.5–36 kbp), can transduce both quiescent and dividing cells, and present a minimal risk of integration of vector DNA into the host [1]. Amongst human adenoviruses, the most widely studied and used in gene therapy preclinical studies is human adenovirus 5 (HAdV-5). Nonetheless, use is hampered by several factors such as the high level of pre-existing neutralizing antibodies against HAdV-5 virions in the clinical population [2,3] and hepatic tropism following intravenous administration, which can lead to acute liver toxicity in humans, non-human primates and rodent models [4,5,6,7].

The high hepatic tropism of HAdV-5 is putatively mediated by the binding of the capsid hexon hypervariable regions (HVRs) of HAdV-5 hexon with blood coagulation factor X (FX) [8], which in turn interacts with heparan sulphate proteoglycans (HSPGs) present on the surface of hepatocytes [9,10], and thus results in virion accumulation in the liver. Moreover, when FX is bound to the virions it also serves as a shield to protect the virus against immune neutralization both in vitro and in vivo. FX binding to HAdV-5 prevents complement activation and binding of C3 to the viral capsid [11]. However, FX shielding is not functionally relevant for some serotypes, since the serotypes HAdV-35 and HAdV-50, which also bind FX, are not neutralized in vitro by mouse serum, even when FX binding is abrogated [12]. Both liver tropism and immune shielding appear to be mediated primarily by the HVRs, specifically HVR loops 5 and 7. In fact, hexon HVRs are highly variable among AdV serotypes and represent the primary determinant of neutralization specificity. Modification of the capsid HVRs of HAdV-5 by genetically exchanging HVR regions or nucleotides encoding specific amino acids of the HAdV-5 HVR5 and HVR7 for those equivalent regions from a non-FX-binding HAdV has proven an effective strategy to ablate the virion:FX interaction in order to study neutralization and liver tropism effects [8,10,13]. Interestingly, FX shielding is not necessary for protection of virions against neutralization in mice lacking either antibodies, C1q or C4 complement molecules, although liver transduction was decreased when administering a mutant HAdV-5 unable to bind FX (termed AdHVR7) [11]. Mouse Immunoglobulin M (IgM) has a pivotal role in triggering the classical complement pathway in vitro, which can lead to neutralization of adenovirus virions [11]. Furthermore, we recently reported that binding of human FX to the HAdV-5 capsid prevents binding of human IgMs but not binding of human IgGs [14]. When HAdV-5 virions were co-incubated with human FX and IgM, there was a reduction in the size shift of the capsid complex compared to when human IgM was added alone. However, the size shift observed when human IgG was incubated with the capsid was equivalent in both the absence and presence of human FX, demonstrating that the binding was FX-independent [14].

Previously, NanoSight particle size tracking was shown to be a useful method to study the direct binding of human IgM and other serum components to HAdV-5 virions [14]. In the studies presented here we conducted similar experiments with murine IgM and serum and demonstrate that IgM isolated from mouse serum only binds HAdV-5 when C3 and C4b were co-isolated as impurities. This guided us to further assess the interactions of HAdV-5 with the different components of the mouse immune system for a better understanding of these complex mechanisms. These results led us to hypothesize that the complement may play a significant role in adenovirus neutralization and that the alternative pathway may possibly lead to IgM-independent neutralization of HAdV-5 in vitro and/or in vivo.

## 2. Material and Methods

### 2.1. Ethics Statement

All animal experiments were approved by the University of Glasgow Animal Procedures and Ethics Committee and Performed under UK Home Office license (PPL 60/4429) in strict accordance with UK Home Office guidelines. Animals were housed under standard 24 h light/dark cycle, with *ad libitum* access to food and drinking water. All efforts were made to minimize suffering.

### 2.2. Cells and Viral Vectors

SKOV3 (human ovarian carcinoma: ATCC^®^ HTB-77 TM) cells were cultured in RPMI-1640 medium (Invitrogen, USA) supplemented with 2 mM L-glutamine (Invitrogen, Paisley, UK), 10% FBS (PAA Laboratories, Leonding, Austria) and 1% penicillin-streptomycin (Invitrogen, Paisley, UK) at 37 °C and 5% CO_2_. HEK-293 cells (human embryonic kidney: ATCC^®^ CRL-1573 TM) were grown in MEM (Invitrogen, USA) with 2 mM L-glutamine, 10% FBS, 1 mM sodium pyruvate and 1% penicillin-streptomycin (Invitrogen, USA) at 37 °C and 5% CO_2_. Vectors (HAdV-5 luc and LacZ [β-galactosidase]; HAdV-5T* luc and LacZ containing the following mutations I421G, T423N, E424S, L426Y, E451Q in HVR7 and T270P and E271G in HVR5 showing reduced FX-binding capacity [8]) were propagated in HEK-293 cells and purified by CsCl gradient centrifugation [15]. Viral particles were determined by micro BCA assay (Life Technologies, Camarillo, CA, USA) using the formula 1 mg protein = 4 × 10^9^ virus particles (vp). Laser-based nanoparticle tracking analysis (NTA) was used to characterize the size of adenoviral particles from pure preparations with Nanosight NTA v2.3 software in a NanoSight LM14 (Malvern Panalytical, Malvern, UK). Plaque forming units (pfu)/mL were calculated by end-point dilution assay [15]. The luc vectors were used for all the in vitro experiments, whereas LacZ vectors were used for the in vivo analyses.

### 2.3. Fluorescent Labelling of HAdV Particles

HAdV-5 and HAdV-5T* were fluorescently labelled using an the Alexa Fluor 532 protein labelling kit (Invitrogen, Paisley, UK) described in detail in [14].

### 2.4. Nanoparticle Tracking Analysis (NTA)

Alexa Fluor 532 labelled HAdV-5 or HAdV-5T* vectors (1 × 10^9^ vp) were incubated at room temperature for 90 min in PBS (10 µL final volume) with physiologically relevant concentrations of purified mouse IgM (1000 µg/mL) (Rockland Immunochemicals, Limerick, PA, USA) and mouse IgG (1000 µg/mL) (Sigma-Aldrich, Gillingham, UK) in the presence or absence of 5 µg/mL human blood coagulation factor X (FX) (Cambridge Biosciences, Cambridge, UK) or with different naïve murine sera (C57BL/6, *Rag2^-/-^*, Ighm^tm1Che^; native or inactivated) in the presence or absence of 40 µg/mL X-bp (25 µL final volume). Human FX has been shown to be able to be substituted by mouse FX [16]. The mutant Ighm^tm1Che^ C57BL/6 mice have B cells that do not secrete IgM, but that express membrane-bound IgM. Fresh serum from C57BL/6, *Rag2^-/-^*, or Ighm^tm1Che^ C57BL/6 mice was separated from whole blood after clotting by centrifugation (10 min, 4 °C, 2000× *g*). Serum inactivation was carried out by 30 min 56 °C incubation. Samples were then diluted in 1.5 mL PBS and injected into the Nanosight LM14 (Malvern Panalytical, Malvern, UK), where the fluorescent virus particle sizes were tracked along time. Results are shown as data from a minimum of three separate experiments with approximately 700 completed tracks.

### 2.5. Serum Neutralization Assay

Human cells were seeded in 96-well plates (1 × 10^4^ cells/well) and incubated overnight at 37 °C and 5% CO_2_. Fresh serum from naïve C57BL/6 mice, *Rag2^-/-^*, or Ighm^tm1Che^ C57BL/6 mice was incubated with 1 × 10^9^ vp of vector (HAdV-5 luc) in a final volume of 50 μL (30 min at 37 °C). The potential interactions with mouse FX were inhibited by the addition of 40 μg/mL X-bp. Samples were then diluted 200-fold in serum-free medium. A total of 1000 vp/cell were added onto cells for 2 h at 37 °C, then media was replaced with fresh media containing 2% FBS, and after further ~16 h at 37 °C cells were rinsed with PBS and harvested by freezing and thawing in Reporter Lysis Buffer (Promega, UK) for both luciferase assay (Promega, Southampton, UK) and protein content measurement (BCA assay) using a VICTOR^TM^ X3 Multilabel Plate Reader (PerkinElmer, Waltham, MA, USA).

### 2.6. Isolation of Proteins Bound to the Virions

50 µg (corresponding to 2 × 10^11^ vp) of each of the adenovirus vectors HAdV-5 and HAdV-5T* was incubated with 250 µL of C57BL/6 mouse serum during 30 min at 37 °C. After that, virion particles and proteins bound to the virus were purified by CsCl gradient ultracentrifugation, as previously described [15]. Briefly, 2 layers of CsCl were placed in an appropriate tube: 4 mL of 1.25 density CsCl and 4 mL of 1.40 carefully added under the other, in order to create the gradient. The mixture of virus and sera was placed on top of the CsCl cushion drop-wise. Tubes were then centrifuged in a SW41 rotor on a Sorvall WX + Ultracentrifuge (Thermofisher, Waltham, MA, USA) for 1.5 h at room temperature. The white band corresponding to the virions and the proteins bound was evident after this ultracentrifugation step was carefully collected by piercing the plastic tube with a needle from the side. As an internal control, a similar tube with only C57BL/6 mouse serum was run in parallel and a wide amount of liquid at a similar level to that of the virus sample was collected to rule out contamination of the sample with serum protein complexes co-migrating with the virus. To remove CsCl, sample buffer was exchanged to 10 mM Tris, 1 mM EDTA pH = 8 buffer (TE 1x) using Amicon 10 kDa filters (Merck, Darmstadt, Germany). Proteins were additionally concentrated by precipitation using cold acetone and resuspended in Laemmli sample buffer. Analysis by SDS-PAGE gel electrophoresis showed no detectable protein in controls, while virus exposed to sera sample showed a similar pattern to that of the virus alone (Supplementary Figure S1).

### 2.7. Liquid Chromatography-Mass Spectrometry (LC-MS/MS)

Five μg of total protein from each sample was loaded into an SDS-PAGE gel and stopped immediately after the samples entered the gel. The gel was stained with InstantBlue Protein Stain (Expedeon, Heidelberg, Germany) for 1 h and destained in 10% acetic acid overnight to visualize the sample bands. Subsequently, each sample was isolated as a unique band and in-gel digestion with trypsin performed as described elsewhere [17]. Peptide samples were then subjected to liquid chromatography (LC) in an Ultimate 3000 RSLCnano Systems (Dionex; Thermofisher, Waltham, MA, USA), being separated on a 50 cm EASY-Spray column (Thermofisher, Waltham, MA, USA) assembled in an EASY-Spray source (Thermofisher, Waltham, MA, USA) and operated at 50 °C. The LC was directly coupled to an Orbitrap Fusion Lumos Tribrid mass spectrometer (Thermofisher, Waltham, MA, USA) for protein identification. Label-free quantification analysis was performed by employing the MaxLFQ algorithm as described elsewhere [18]. Peptide and protein identifications were filtered using a 1% FDR. All Label-free quantification (LFQ) MS analyses were performed using two complete biological replicates of each of the conditions being compared. Amounts of C3, C4b and IgM were calculated as LFQ of the protein of interest normalised to adenovirus 5 hexon protein’s LFQ.

### 2.8. In Vivo Experiments

For in vivo experiments, male C57BL/6 inbred mice and *Rag2^-/-^* (B6(Cg)-*Rag2^tm1.1Cgn^*/J) knockout mice aged between 10 to 12 weeks were used. A total of 4 × 10^12^ vp/kg of HAdV-5 LacZ or HAdV-5T* LacZ in 150 μL Dulbecco’s phosphate-buffered saline (DPBS) were administered via the tail vein. Animals were humanely killed 48 hr post-injection, perfused with DPBS, and internal organs (liver and spleen) were either collected and fixed in 2% PFA overnight at 4 °C, frozen in liquid nitrogen or embedded in Optimal Cutting Temperature compound (OCT) in cryomolds (Tissue-Tek, Torrance, CA, USA) and frozen at −80 °C. DNA extraction was carried out from frozen liver and spleen samples using the QIAamp DNA mini kit (QIAGEN, Hilden, Germany) according to the manufacturer’s instructions. Viral genome content was quantified by SYBR green-based qPCR [8]. Protein was extracted from frozen liver (~25 mg) and spleen (~10 mg). Samples were placed in a 2 mL microcentrifuge tube containing 1 mL of lysis buffer provided in the anti-β-galactosidase enzyme-linked immunosorbent assay (ELISA) kit (Roche, Welwyn Garden City, UK) and two stainless steel beads (3 mm). Tissues were homogenized using a TissueLyser II (QIAGEN, Manchester, UK) for 1 min at 25 mHz twice, tissue lysates were subjected to centrifugation at 24,000× *g* for 1 min at 4 °C and supernatant was transferred to a clean 1.5 mL microcentrifuge tube for storage at −80 °C. β-galactosidase content was quantified by the anti-β-galactosidase ELISA kit (Roche, Basel, Switzerland) according to the manufacturer’s instructions and values were normalized by the total protein concentration in the samples measured by the BCA Protein assay (Thermofisher, Waltham, MA, USA ). Immunohistochemistry analyses of β-galactosidase were carried out on 6 μm frozen liver and spleen sections [13]. Rabbit anti-β-galactosidase primary antibody and Alexa Fluor 488-conjugated goat anti-rabbit IgG secondary antibody were used. Nuclei were counterstained with DAPI. X-Gal staining was performed in liver and spleen samples previously fixed in 2% PFA.

### 2.9. Detection of IgM and IgG

Commercially available ELISA kits (Abcam, Cambridge, UK) were used according to the instructions of the manufacturer for the detection of IgM and IgG in murine serum. IgG depletion was carried out by Dynabeads Protein G (Thermofisher, Waltham, MA, USA).

### 2.10. Statistical Analysis

Statistical significance was calculated using one-way ANOVA followed by Tukey post hoc test with GraphPad Prism 4.00 (GraphPad Software, San Diego, CA, USA). *p*-values of 0.05 or below were considered statistically significant. Each in vitro result presented is data from a minimum of three separate experiments with at least four experimental replicates per group. LC-MS experiments were performed twice to assure reliability of the results obtained. In vivo experiments were performed with a minimum of three animals per group (*n* = 5–6 for HAdV treated groups, *n* = 3 for DPBS groups). Values are shown as the mean ± SEM and unpaired Student’s *t*-test was applied.

## 3. Results

### 3.1. Differential Binding of Different Sources of IgM

**Figure 1:**
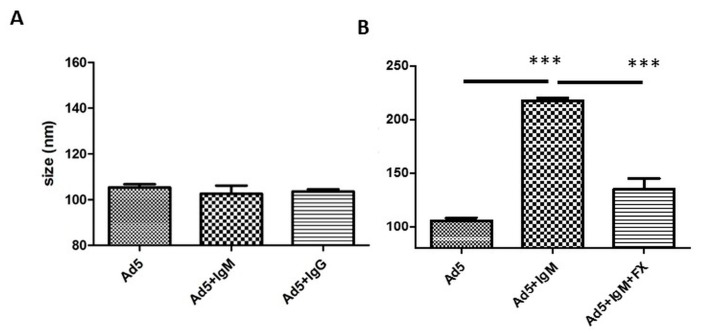
Mouse IgM does not bind to HAdV-5 in the absence of complement molecules. (**A**) Commercially available purified IgM and IgG are not able to bind to HAdV-5. Alexa Fluor 532-labelled HAdV-5 vector was incubated at room temperature for 90 min in PBS with physiologically relevant concentrations of purified mouse IgM and mouse IgG. Samples were diluted in PBS and injected into the Nanosight. Data from a minimum of three separate experiments with ~700 completed tracks are shown as the mean ± SEM. One-way ANOVA and Tukey’s post hoc test applied. (**B**) Non-commercial mouse IgM (provided by Dr. A.P. Byrnes; “home-purified murine IgM”) binds to HAdV-5 in a FX-dependent manner, similarly to human IgM [14]. Further investigations showed that these IgMs were contaminated by complement components. Alexa Fluor 532-labelled HAdV-5 vector was incubated at room temperature for 90 min in PBS with physiologically relevant concentrations of mouse IgM in the presence or absence of human FX, then diluted in PBS and injected into the Nanosight LM14. Data from a minimum of three separate experiments with ~700 completed tracks are shown as the mean ± SEM. One-way ANOVA and Tukey’s post hoc test applied. *** *p* < 0.001 vs. matched control or IgM conditions.

Using NTA, we aimed to first analyse the potential binding of murine IgM and IgG to HAdV-5. There was no size shift of the HAdV-5 particles observed following incubation of virions with commercially available purified murine IgM and IgG (Figure 1A). However, incubation with a purified murine IgM (kindly provided by Dr. A.P. Byrnes, Maryland, USA; herein called “home-purified murine IgM”) did show a size shift, suggesting that binding of serum components to HAdV-5 occurred in a FX-dependent manner (Figure 1B), in similarity to that previously observed for human IgM [14]. These surprising results pointed to a potentially different nature of home-purified and commercial murine IgM and suggested the possibility that IgM might not be able to bind HAdV-5 under certain conditions.

### 3.2. Differential Binding of Serum Components to HAdV-5 Virions

**Figure 2:**
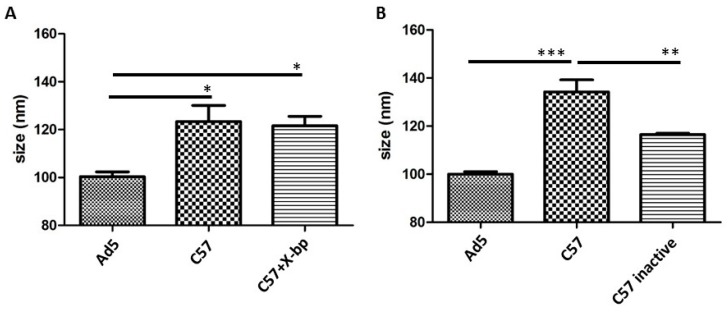
HAdV-5 binds to murine serum components in a FX-independent manner, but in a complement-dependent way. (**A**) HAdV-5 binds to immunocompetent murine serum components regardless of the presence of FX. Alexa Fluor 532-labelled HAdV-5 was incubated at room temperature for 90 min in murine sera -/+ X-bp. Samples were diluted in PBS and injected into the Nanosight LM14. Data from a minimum of three separate experiments with ~700 completed tracks are shown as the mean ± SEM. One-way ANOVA and Tukey’s post hoc test applied. * *p* < 0.05 vs. matched control. (**B**) Significant particle size drop was observed incubating HAdV-5 in heat-inactivated immunocompetent murine serum compared to active serum. Alexa Fluor 532-labelled HAdV-5 was incubated at room temperature for 90 min in murine sera. Serum inactivation was carried out by a 30 min 56 °C incubation. Samples were diluted in PBS and injected into the Nanosight. Data from a minimum of three separate experiments with ~700 completed tracks are shown as the mean ± SEM. One-way ANOVA and Tukey’s post hoc test applied. ** *p* < 0.01, *** *p* < 0.001 vs. matched control or inactive serum.

**Figure 3:**
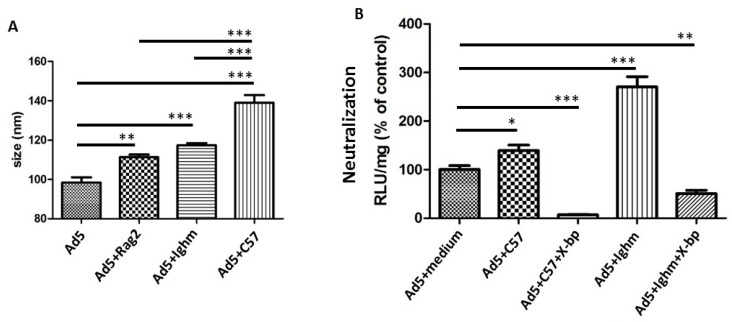
Murine IgM is not crucial for neutralization in vitro. (**A**) HAdV-5 binds to *Rag2^-/-^*, Ighm^tm1Che^ and wild type (WT) (immunocompetent C57BL/6) serum components. *Rag2^-/-^* serum lacks mature T and B lymphocytes, while Ighm^tm1Che^ lacks only soluble IgM. Alexa Fluor 532-labelled HAdV-5 was incubated at room temperature for 90 min in murine sera. Samples were diluted in PBS and injected into the Nanosight LM14. Data from a minimum of three separate experiments with ~700 completed tracks are shown as mean ± SEM. One-way ANOVA and Tukey’s post hoc test applied. ** *p* < 0.01, *** *p* < 0.001 vs. matched control or WT serum. (**B**) Ighm^tm1Che^ serum is able to neutralize HAdV-5 in the absence of FX. Human cells were seeded in 96-well plates. Then different murine sera (Ighm^tm1Che^ or wild type) were incubated with HAdV-5 in the presence or absence of X-bp. A total of 1000 vp/cell were added onto cells, and after further ~16 h at 37 °C, cells were harvested in Reporter Lysis Buffer (Promega, UK) for luciferase assay and protein content measurement using a VICTOR^TM^ X3 Multilabel Plate Reader. Data from a minimum of four separate experiments are shown as mean ± SEM. One-way ANOVA and Tukey’s post hoc test applied. * *p* < 0.05, ** *p* < 0.01, *** *p* < 0.001 vs. matched controls.

We next investigated the binding of proteins present in mouse serum to HAdV-5 virions. Incubation of HAdV-5 with immunocompetent (C57BL/6) murine serum showed that serum components are able to bind to the virion at similar levels in both the presence or absence of the FX inhibitor X-bp (Figure 2A, compare the “C57” condition to “C57 + X-bp”). X-bp is a protein isolated from the venom of *Trimeresurus flavoviridis* that belongs to the C-type lectin superfamily and binds to the GLA domain of FX, inhibiting its interaction with the hexon of HAdV-5 [19]. The fact that X-bp could indeed inhibit FX binding in our experimental setting was confirmed by performing neutralization assays in parallel (Figure 3B, 3rd column). Since Figure 1B showed that binding of murine IgM to virions was greatly reduced in the presence of FX, we suggest the size shift observed in Figure 2A in the presence/absence of FX binding (+/- X-bp) may be predominantly due to complement components binding to the virion. Moreover, the size shift observed was complement-dependent, since a significant reduction in particle size was observed for HAdV-5 incubated with heat-inactivated immunocompetent murine serum (heat is reported to inactivate the complement [11]), compared to that with untreated serum (Figure 2B). These results suggested a potential binding of serum proteins to the virions both in the presence and absence of FX, with a substantial contribution of complement factors.

To further characterize which serum immunoglobulins might bind to HAdV-5, sera from immunocompetent and two types of immunoglobulin-deficient mice was utilised. Mouse strains were the B and T cell deficient *Rag2^-/-^* mice in which all immunoglobulin isotypes are absent and Ighm^tm1Che^ mice, in which B cells cannot secrete IgM [20] but are competent to secrete all other immunoglobulin isotypes. Comparison of the size shift between virions incubated with sera from immunoglobulin-deficient mice (*Rag2^-/-^*, Ighm^tm1Che^ C57BL/6) to that of immunocompetent mouse sera (C57BL/6) showed that serum components from all types of sera were able to bind to HAdV-5 virions (Figure 3A, comparing the last three bars with HAdV-5). The binding observed when HAdV-5 was incubated with the immunoglobulin-deficient sera may be due to complement biding directly to the viral capsid. This was consistent with the aforementioned results obtained with heat-inactivated immunocompetent murine serum (Figure 2B, compare “C57 inactive” to “C57”). However, there was a significant increase in particle size when HAdV-5 was incubated with the immunocompetent C57BL/6 serum (Figure 3A, bar 4), in comparison to *Rag2^-/-^* or Ighm^tm1Che^ C57BL/6 sera (Figure 3A, bars 2 and 3, respectively). This size shift may be due to the binding of immunoglobulins, presumably IgM. Together the results obtained suggest that complement could bind HAdV-5 in a FX-independent manner while pure IgM alone cannot bind. While binding was FX-independent, virion neutralization was shielded by FX. This implied that the mechanism by which FX protected HAdV-5 might not be only relying on impeding the binding of immune response proteins to the capsid.

### 3.3. Murine IgM and IgG are not Necessary for Neutralization In Vitro

Both Ighm^tm1Che^ C57BL/6 and immunocompetent sera enhanced virus transduction (Figure 3B, bars 4 and 2, respectively) as a result of mFX shielding the virions and thus preventing virus neutralization and also facilitating cell attachment [11]. Incubation with Ighm^tm1Che^ C57BL/6 serum, which does not contain IgM, not only enhanced transduction but also showed a greater transduction than that with immunocompetent sera (Figure 3B, compare bar 4 to bar 2). Interestingly, when HAdV-5 was incubated with X-bp to inhibit mFX binding, Ighm^tm1Che^ C57BL/6 serum neutralized HAdV-5, similarly to that observed with immunocompetent mouse serum (Figure 3B, bars 5 and 3, respectively) suggesting that murine IgM was not necessary for neutralization in vitro.

**Figure 4:**
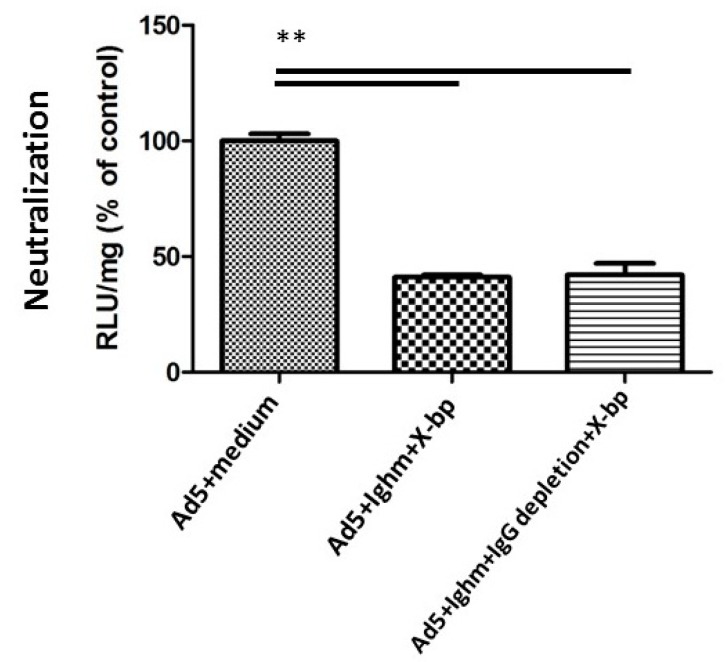
IgG depletion does not inhibit serum neutralization. This assay was performed to determine whether IgG was responsible for triggering neutralization in Ighm^tm1Che^ serum. Human cells were seeded in 96-well plates. Then Ighm^tm1Che^ murine serum (with or without depletion of IgG by Dynabeads Protein G) was incubated with HAdV-5 for 30 min at 37 °C. A total of 1000 vp/cell were added onto cells, and after further ~16 h at 37 °C cells were harvested for luciferase assay and protein content measurement using a VICTOR^TM^ X3 Multilabel Plate Reader. Data from a minimum of four separate experiments are shown as mean ± SEM. One-way ANOVA and Tukey’s post hoc test applied. ** *p* < 0.01 vs. matched controls.

Next, IgG was depleted from Ighm^tm1Che^ C57BL/6 mouse serum, and a serum neutralization assay was performed to determine whether IgG was responsible for triggering neutralization (Figure 4). Depletion of IgG did not inhibit serum neutralization, thus showing that IgG was not necessary for this process. The absence of soluble IgM in Ighm^tm1Che^ C57BL/6 serum or IgG in IgG-depleted Ighm^tm1Che^ C57BL/6 serum was confirmed using ELISA, where the immunoglobulin concentration was below the detection limit. Therefore, both IgM and IgG were found to be unnecessary for the FX-dependent neutralisation of HAdV-5.

### 3.4. IgM, C3 and C4b Bind to HAdV-5 in the Presence of FX

**Figure 5:**
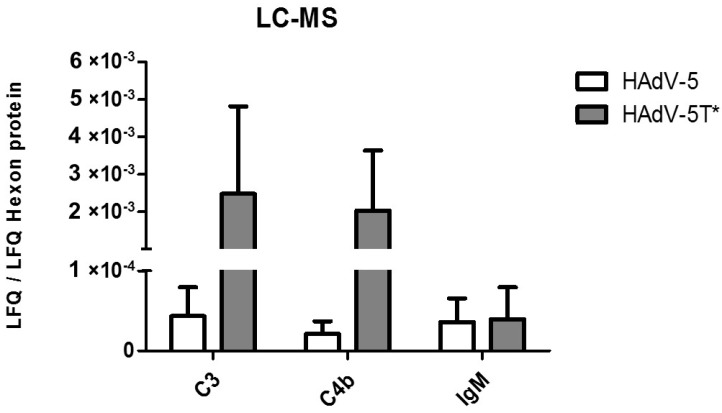
Binding of complement proteins C3 and C4b is substantially higher on HAdV-5T* virions. A total of 50 µg (corresponding to 2 × 10^11^ vp) of HAdV-5 or HAdV-5T* were incubated with 250 µL of C57BL/6 mouse sera at 37 °C for 30 min. Next, virion particles and proteins bound to the virus were purified by CsCl gradient. The mixture of viral and serum proteins was loaded into an SDS-PAGE gel and subjected to electrophoresis until immediately after the samples entered the gel. Following in-gel tryptic digestion, peptide samples were analysed by LC-MS/MS in an Orbitrap Fusion Lumos Tribrid mass spectrometer (Thermo Fisher Scientific) for protein identification. Label-free quantification analysis were performed by employing the MaxLFQ algorithm. Peptide and protein identifications were filtered using a 1% FDR. All LFQ MS analyses were performed using two complete biological replicates of each of the conditions being compared. Amounts of C3, C4b and IgM were calculated as LFQ of the protein of interest normalised to adenovirus 5 hexon protein’s LFQ. LFQ: label-free quantification.

The NTA results showed that commercially available human IgM and non-commercial mouse IgM produced a shift in the HAdV-5 size, while commercially available murine IgM failed to do so (Figure 1). Next the purity of the different immunoglobulins were therefore analysed by LC-MS. These data demonstrated that human (Sigma, USA) and non-commercial mouse IgM were, among other proteins, contaminated with C3, C4b and C4b-binding protein, while the commercially available murine IgM (Rockland Immunochemicals, USA) was free of these or contained substantially less (Supplementary Table S1). These observations suggest that IgM was only able to bind HAdV-5 when complement proteins were present. Next, LC-MS was used to assess which serum components bound to adenovirus vectors (Supplementary Table S2). It was found that complement components (C3, C4b) and IgM bound to HAdV-5 (Figure 5), confirming the NTA data (Figure 2). When HAdV-5T* (a HAdV-5 vector which is unable to bind to either murine or human FX [8]) was tested, the amounts of bound C3 and C4b detected were higher compared to those bound to HAdV-5 (fold changes: C3 = 40.2 ± 20.3; C4 = 87.8 ± 10.6), whereas IgM binding to HAdV-5T* was not different to that observed for HAdV-5 (Figure 5). These results provide evidence for the binding of IgM, C3 and C4b to HAdV-5 even in the presence of FX. Moreover, the binding of C3 and C4b was higher for HAdV-5T*, a mutant vector that cannot bind FX.

### 3.5. HAdV-5T* Is Partially Neutralized in Immunoglobulin-Deficient Mice

**Figure 6:**
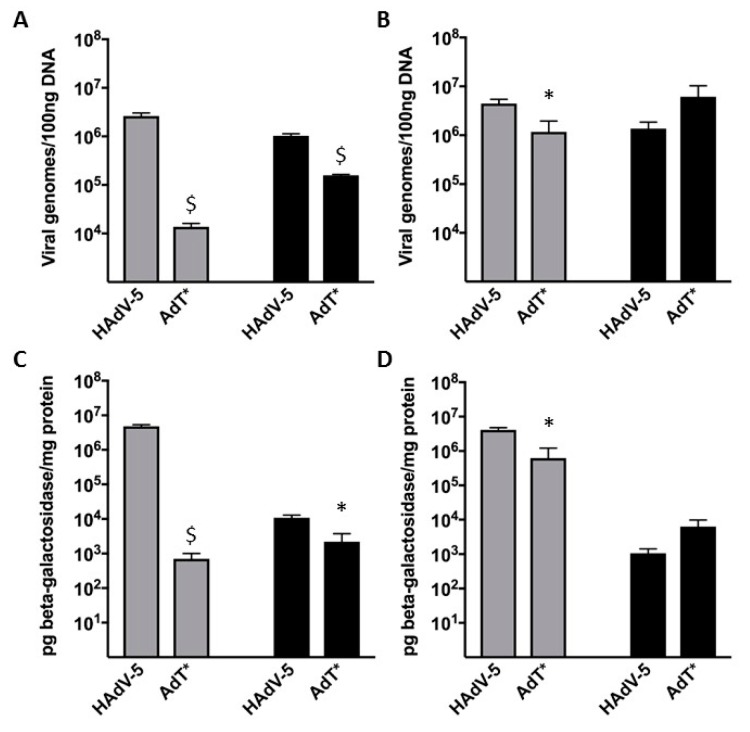
Accumulation of adenoviral genomes and transduction levels in liver and spleen. In C57BL/6 mice, the transduction levels and number of genomes detected in the liver and spleen following HAdV-5T* administration were significantly lower than those of HAdV-5. Interestingly, a similar pattern was observed in *Rag2^-/-^* mouse livers, indicating that neutralization of HAdV-5T* still occurred, even in the absence of mature B and T cells. C57BL/6 (**A** and **C**) or *Rag2^-/-^* (**B** and **D**) were administered 1 × 10^11^ vp of HAdV-5 LacZ or HAdV-5T* LacZ by intravascular delivery and euthanized and perfused with DPBS 48 h post-injection. Viral genome content was quantified by SYBR green-based QPCR analysis in liver (grey bars) and spleen (black bars) (**A**–**B**). β-galactosidase expression was quantified by ELISA in liver and spleen and normalised to total mg of protein (**C**–**D**). n = 5–6. Values are expressed as the mean ± SEM. Unpaired Student’s *t*-test applied. * *p* < 0.05 vs. HAdV-5, $ *p* < 0.01 vs. HAdV-5.

**Figure 7:**
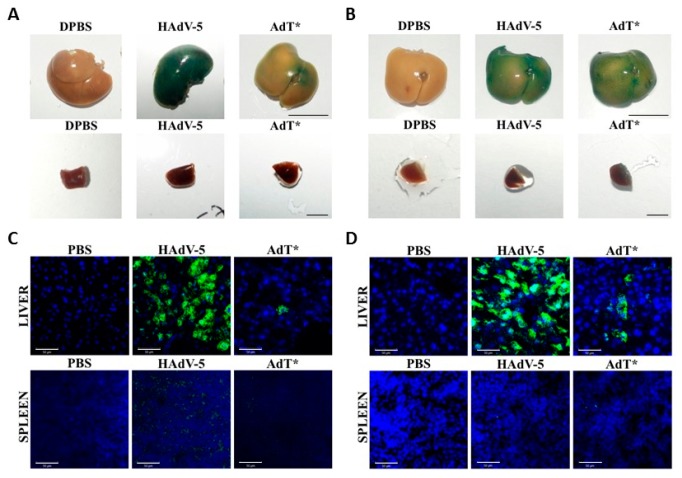
β-galactosidase expression in mouse liver and spleen. C57BL/6 (**A** and **C**) or *Rag2^-/-^* (**B** and **D**) mice were administered 1 × 10^11^vp of HAdV-5 LacZ, HAdV-5T* LacZ or DPBS by intravascular delivery and euthanized and perfused with DPBS 48 h post-injection. (**A** and **B**) Representative images of X-Gal staining of liver and spleen fixed in 2% paraformaldehyde are shown. Scale bars 1 cm. (upper panels) or 0.25 cm (lower panels). (**C**) and (**D**) Immunohistochemistry analysis of β-galactosidase on 6 μm frozen liver and spleen sections. Rabbit anti-β-galactosidase primary antibody and Alexa Fluor 488-conjugated goat anti-rabbit IgG secondary antibody (green) were used. Nuclei were counterstained with DAPI (blue). n = 3 mice analysed per group with 1 technical replicate. Representative merged images are shown. Magnification 40×. Scale bars 50 μm.

Since IgM and IgG were not crucial for neutralization in vitro, next in vivo experiments were conducted to further analyse these findings. C57BL/6 or *Rag2^-/-^* mice were administered HAdV-5 LacZ or HAdV-5T* LacZ vectors. In C57BL/6 mice, both transduction levels and the number of genomes detected in the liver and spleen following HAdV-5T* administration were significantly lower than those detected for HAdV-5 (Figure 6A,C). Immunohistochemical analysis of β-galactosidase on liver and spleen sections confirmed these findings (Figure 7A,C). These data suggested that either the lack of HAdV-5T* binding to FX, and/or shielding of the capsid from complement by FX, leads to virus neutralization. Interestingly, a similar pattern (reduced transduction levels and number of genomes mediated by HAdV-5T* compared to HAdV-5) was observed in *Rag2^-/-^* mouse livers (Figure 6B,D and Figure 7B,D), indicating that partial neutralization of HAdV-5T* still occurred, even in the absence of mature B and T cells. There was no significant difference in the transduction levels or number of genomes detected from each vector in the spleen of *Rag2^-/-^* mice (Figure 6B,D and Figure 7B,D). The differential splenic tropism for FX-binding-ablated HAdV-5 that has been formerly reported [13] might have attenuated the effect of the partial neutralization in *Rag2^-/-^* mice spleens. These results altogether suggest that immunoglobulins are not required for partial neutralization of HAdV-5 in vivo.

**Figure 8:**
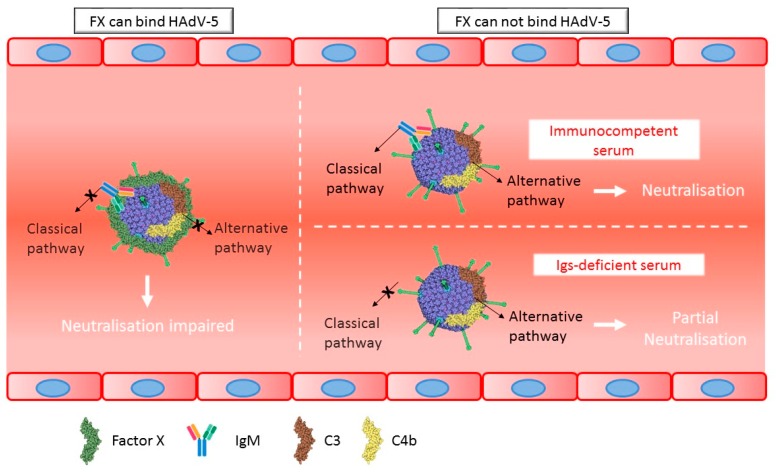
Schematic model of the interactions of HAdV-5 with the host immune system. Neutralization of HAdV-5 in naïve mice occurs if FX binding to the virions is ablated. In immunocompetent mice both classical and alternative complement pathways are activated, while in immunoglobulin-deficient mice partial neutralization is caused by the alternative pathway.

In summary, the data generated suggest that (1) immune response proteins can bind HAdV-5 virions in naïve mice but neutralization only occurs if FX is absent or the virion is unable to bind FX, (2) purified mouse IgM antibodies bind to HAdV-5 only in the presence of complement components and (3) in serum from immunocompetent mice both classical and alternative complement pathways are activated, while in serum from immuno-deficient mice with absent immunoglobulins, the alternative complement pathway mediates partial neutralization (Figure 8).

## 4. Discussion

In this study, we demonstrated that murine IgM is not pivotal for either in vitro or in vivo HAdV-5 neutralization. It was observed that IgM alone does not bind to HAdV-5, however IgM does bind in the presence of specific complement proteins. Interestingly, these binding events were observed even in the presence of FX. The in vitro and in vivo data suggest that serum proteins other than IgM or IgG might also trigger the immune response, implying the importance of the alternative complement pathway in HAdV-5 neutralisation.

It has been previously reported that HAdV-5 specifically binds to murine and human FX, and that the capsid:FX interaction inhibits the neutralization mediated by IgM and complement components [11,21]. A previous study based on particle size tracking demonstrated that purified human IgM and IgG were able to bind to HAdV-5 virions, with IgM only binding in a FX-dependent manner [14]. Given the pivotal role for murine IgM in virus neutralization both in vivo and in vitro [11], it was expected that murine IgM would behave in a similar fashion to human IgM. However, the results here revealed that while non-commercial IgM was able to bind to HAdV-5 virions, commercially purified murine IgM showed no binding (Figure 1). Interestingly, LC-MS analysis showed that non-commercial IgM preparations contained C3, C4b and C4b-binding protein (Supplementary Table S1). Similarly, commercially available human IgM, which we have previously proved to be able to bind HAdV-5 [14], was also analysed by LC-MS and found to be contaminated with C3, C4b and C4b-binding protein. These findings suggest that either murine IgM is not able to bind to HAdV-5 virions in the absence of complement molecules or that it is actually the complement proteins that bind to the virions. Since C3 and C4b can covalently bind to pathogens to trigger an immune response [22], it might also be possible that C4b binds to C3, forming a complex that protects C3 from inactivation. Further studies with completely purified human IgM would clarify whether human IgM is able to bind to the virion’s surface without requirement for the presence of complement molecules.

Analysing the dataset obtained by nanoparticle tracking of HAdV-5 incubated with C57BL/6 sera, we demonstrated that serum components bind to the virions in a FX-independent manner and that this binding is greatly reduced by heat inactivating the serum (Figure 2). Interestingly, murine sera from different immunoglobulin-deficient mice strains (*Rag2^-/-^*, Ighm^tm1Che^), behaved in a similar manner, suggesting that at least some of the complement molecules directly attach to HAdV-5 capsids without requiring the presence of immunoglobulins (Figure 3). Furthermore, HAdV-5 was neutralized by immunocompetent C57BL/6 serum in the absence of FX, as previously demonstrated [11,21]. However, regardless of the presence of FX, some of the molecules involved in the immune response were able to bind to the virions. These data were further confirmed by LC-MS analysis, which demonstrated that complement molecules and IgM can bind to HAdV-5, even in the presence of FX. When comparing HAdV-5 with the mutant adenoviral vector HAdV-5T*, which cannot bind to FX [8], substantially higher amounts of C3 and C4b were found bound to HAdV-5T* in comparison to HAdV-5. In contrast, there was no variation observed for IgM binding (Figure 5). Together, these data suggest that FX may share binding sites with complement proteins, in agreement with previous data [11], but that complement can also bind to alternative sites on the surface of the HAdV-5 virion. This may mean that the mechanism by which FX protects HAdV-5 might not only rely on impeding the binding of immune response proteins to the capsid. In the presence of FX, a lower amount of complement molecules would bind to the virions, perhaps due to competition for binding sites, steric constraints, or key complement molecules also becoming inactivated, in turn inhibiting neutralization. The presence of C3 and C4b on the virus after CsCl isolation without performing any cross-linking step points to a probable covalent binding of both C3 and C4b to the viral capsid. In the case of C3, this might indicate that the alternative complement pathway can be triggered by direct binding of C3b to the viral capsid and would explain why IgM is not required for HAdV-5 neutralisation. However binding of C3b could also occur as part of the C3 amplification loop [23]. Binding of C4b, on the contrary, might point to the activation of the classical pathway, which could be occurring when IgM or potentially other immunoglobulins are present. Binding of C4b to the viral capsid has only been reported very recently and has been demonstrated to impair capsid disassembly and virus entry into the cytosol [24]. This would imply both the classical and alternative complement pathways could operate in an immune response against HAdV-5 when injected intravenously. Alternatively, as aforementioned, it could also be possible for C4b to bind to C3b and not to the capsid, protecting C3 from inactivation and therefore also contributing to the activation of the alternative pathway. However, recent evidence has shown the importance of direct binding of C4b to the capsid of HAdV-5 [24].

Previous findings with human serum showed a significant shift in virus particle size following incubation of virions with human sera samples [14]. However, only in half of the human serum samples was this shift further increased in the presence of X-bp, which blocks the binding of FX to the hexon [8]. Thus, FX prevented binding of human sera components to HAdV-5 virions only in a subset of human samples [14]. In contrast, our findings with murine serum did not show a further increase in particle size in the presence of X-bp. The finding that heat inactivated C57BL/6 serum does not bind to HAdV-5 and does not neutralise the virus agrees with previous studies [11,21]. The difference in the size of HAdV-5 incubated with three different types of sera (*Rag2^-/-^*, Ighm^tm1Che^, C57BL/6) could be explained by the fact that *Rag2^-/-^* and Ighm^tm1Che^ mice do not produce IgM [20]. IgM has an approximate size of 30 nm in diameter, corresponding to the size shift observed by NTA. Unfortunately, exploring the binding of FX to the virions by means of NTA was impossible since the size of FX is 9 nm, below the detection limit of the NanoSight (10 nm) [25].

*Rag2^-/-^* serum was previously reported to not neutralise HAdV-5 [11]. However here, the Ighm^tm1Che^ serum was able to neutralize the virus in vitro when shielding of the virus by FX binding was abolished, suggesting that IgM is not crucial for in vitro HAdV-5 neutralization. It was reported recently that other immunoglobulin molecules may trigger complement activation, and both human IgM and IgG have been used for rescuing immune responses in *Rag2^-/-^* serum [26]. When IgG was depleted from Ighm^tm1Che^ serum in order to investigate if IgG could be the factor that was triggering the observed immune response, IgG depleted serum was still able to neutralize HAdV-5 with comparable efficiency to the non-depleted serum (Figure 4). This could be explained by two possibilities: (1) other immunoglobulin molecules could also trigger the immune response through the classical complement pathway, since the depletion method used is not able to deplete IgA, IgD and IgE from the serum or (2) C3b directly binds to the capsid and in the absence of IgM, could neutralize the virus by activating the alternative complement pathway. Future work will be necessary to fully decipher the exact mechanisms involved in neutralization of HAdV-5 virions.

Although *Rag2^-/-^* serum is not able to neutralize HAdV-5 at all in vitro [11,21], in vivo experiments showed that HAdV-5 is partially neutralized in *Rag2^-/-^* mice (Figure 6 and Figure 7). This is in contrast with previous findings [11], suggesting that immunoglobulins are not required for neutralization of HAdV-5 in vivo. The unexpected observation regarding the adenovirus neutralizing ability of immunoglobulin-deficient mice sera (which fail to generate mature T and B cells) suggests to the presence of a defense mechanism predominantly utilizing the complement cascade.

In summary, mouse IgM is not crucial for in vitro and in vivo HAdV-5 neutralization. Pure IgM alone is not able to bind to HAdV-5 but instead needs the presence of complement molecules. IgM and complement components are able to bind to HAdV-5 regardless of the presence or absence of FX. When FX is shielding the virion, IgM and complement components seem to bind to alternative capsid sites, but FX is able to protect the virion from the immune response. Exposure of complement to heat renders it unable to bind to HAdV-5 virions. In the studied conditions, complement seems to be more crucial than antibodies for successful neutralization to take place. Experiments with Ighm^tm1Che^ serum suggest that immunoglobulins other than IgM or IgG may also trigger the complement cascade in vitro. In vivo, it seems that immunoglobulins are not required for neutralization of HAdV-5. All these findings point to crucial importance for the alternative complement pathway in HAdV-5 neutralisation and show that FX does not completely ablate complement binding but rather impedes its efficient activation. As discussed earlier, the use of HAdV-5 in human gene therapy is hampered by different factors such as the high level of pre-existing neutralizing antibodies [2,3] and its hepatic tropism, which can lead to acute liver toxicity [4,5,6,7]. The data presented here draw attention to the fact that HAdV-5 vectors unable to bind FX (for example: those that have been mutated to avoid hepatic tropism) might be neutralized even in immunoglobulin-deficient individuals. Thus, this should be taken into account for engineering novel adenoviral vectors so as to protect them against the alternative pathway of the complement cascade. These facts should also be considered when designing gene therapies using HAdV-5 vectors, in which temporary suppression of the complement system might be beneficial.

## Supplementary Materials

The following are available online at https://www.mdpi.com/1999-4915/11/7/616/s1, Figure S1: SDS-PAGE gel electrophoresis of HAdV-5 incubated in murine serum. The samples were collected from CsCl cushion. The analysis showed no detectable protein in controls, while virus exposed to serum showed a similar pattern to that of the virus alone, Table S1: Intensity of IgM and complement peptides measured by LC-MS in the different IgMs. The values in the body of the table are LFQ (standardized unit for relative quantification of proteins identified by Mass Spectrometry), Table S2: List of the proteins identified by LC-MS experiments.
