# Supplementary material for: Human Adenovirus Serotype 5 Is Sensitive to IgM-Independent Neutralization In Vitro and In Vivo
Source: Viruses. 2019 Jul 5;11(7):616. doi: 10.3390/v11070616 (PMC6669743; doi:10.3390/v11070616)
Supplement: Supplementary file 1 [file viruses-11-00616-s001.zip › supplementary table 1.docx]

|  | **IgM** | **C3** | **C4b** | **C4-binding protein** |
| --- | --- | --- | --- | --- |
| **Human IgM (Sigma)** | 2,21E+11 | 7,03E+08 | 2,92E+08 | 6,29E+08 |
| **Mouse IgM (Rockland)** | 2,00E+11 | 0,00E+00 | 9,83E+05 | 0,00E+00 |
| **Mouse IgM (non-commercial)** | 9,76E+10 | 5,75E+08 | 8,87E+06 | 3,72E+07 |
