# Supplementary figures and images for: Human Adenovirus Serotype 5 Is Sensitive to IgM-Independent Neutralization In Vitro and In Vivo
Source: Viruses. 2019 Jul 5;11(7):616. doi: 10.3390/v11070616 (PMC6669743; doi:10.3390/v11070616)

## Slide 1
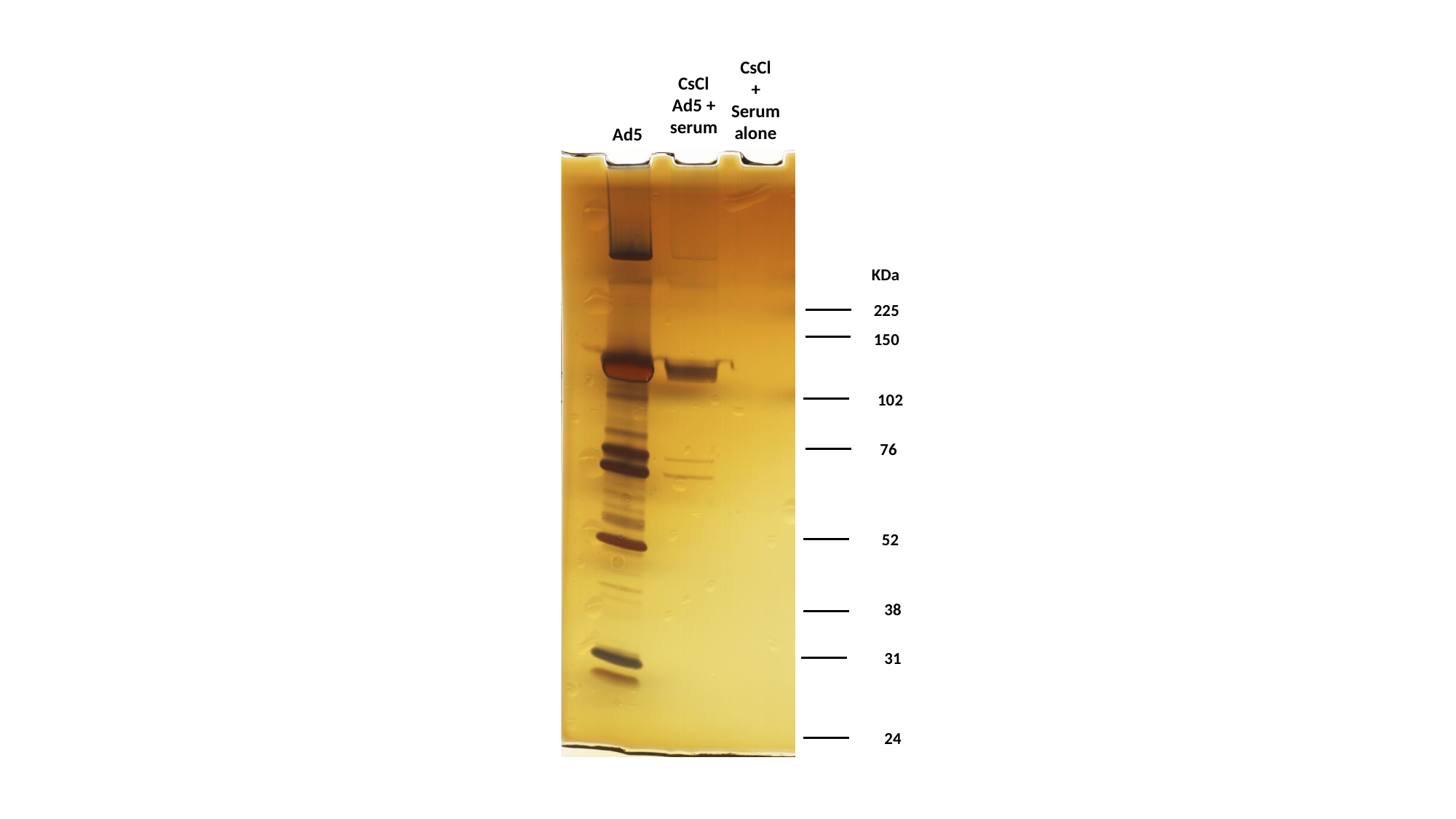

CsCl
+
Serum alone
CsCl
Ad5 + serum
Ad5
KDa
225
150
102
76
52
38
31
24

Supplement: Supplementary file 1 [file viruses-11-00616-s001.zip › Supplementary figure 1.pptx]
